# Physiological interpretations of radiographic findings on malformations of small veins: seriality of cisterns, communications to systemic veins and relationship to muscles

**DOI:** 10.1258/phleb.2012.011137

**Published:** 2014-02

**Authors:** Kazushi Kishi, Nobuo Morita, Tomoaki Terada, Morio Sato, Tetsuo Sonomura

**Affiliations:** * Department of Radiation Oncology, Tumor Center, Wakayama Medical University Hospital, 811-1 Kimiidera, Wakayama City 641-8510; † Department of Oral and Maxillary Surgery, Wakayama Rosai Hospital, 93-1, Kinomoto; ‡ Department of Neurosurgery, Wakayama Rosai Hospital, 93-1, Kinomoto, Wakayama City 640-8453; § Department of Radiology, Wakayama Medical University, 811-1 Kimiidera, Wakayama City 641-8510, Japan

**Keywords:** venous malformations, haemangioma, fluoloscopy, cisternography, draining vein, physiology, rheology, sclerotherapy

## Abstract

**Objectives:**

To re-evaluate the fluoroscopic findings of venous malformation by cine mode cisternography.

**Methods:**

Using direct injection cine-cisternography, we studied 49 venous malformation lesions in the head and neck of 30 patients who were scheduled to undergo ethanol sclerotherapy. The diameter of definitively measurable 46 lesions was 21.7 ± 10.5 mm (mean ± SD, range: 6.0–48.0 mm). The injection was continued until the draining veins were clearly observed. Outflow communications between cisterns and systemic veins were classified into Type 1, no visible drainage; Type 2, draining into a normal venous system; and Type 3, with abnormally ectatic draining veins. The topological relationships of the lesions to surrounding structures were addressed using computed tomography, magnetic resonance imaging or ultrasonogram. Treatment results were evaluated.

**Results:**

The direct injection cine-cisternography showed the typical ‘bunch of grapes’ pattern, and revealed serial cisternal, followed by the appearance of outflow/draining veins in all lesions. There were no Type 1, 47 Type 2 and two Type 2 outflow pattern. Satellite lesions emerged via the communicating veins in six lesions. Of the all 49 lesions, 48 were located in or on the muscle fascia. Sclerotherapy was safely completed in all Type 2 lesions with satisfactory results, but for the Type 3 lesions treatment was limited to be partial to avoid complications.

**Conclusions:**

The present study suggested that communications from venous malformation to the systemic vein are fluoroscopically confirmable. These radiographic findings were thought explainable in relation to developmental nature or facilitating process of venous malformation.

## Introduction

Venous malformation is a low-flow type vascular malformation and is generally characterized by multiple ectatic vascular cavities filled with blood, found most commonly in the head, neck and extremities.

The characteristic radiographic appearance of multiple ectatic cisterns is termed the ‘bunch of grapes’ sign on direct injection cisternography,^1–4^ which enables the distinction of venous malformations from other types of low-flow malformations and varicose veins.^5^ Another characteristic clinical finding of venous malformations is observed as emergence of a cheek or perioral mass during mastication.

Previous radiographic observations on venous malformations^6,7^ report that approximately one-third of venous malformations has no recognizable communications with systemic vessels.^6,7^ However, it is generally considered that the venous malformation lesion is an embryonic vascular structure remnant along the venous system development (bud theory).^1–4^ According to this theory its outflow is all connected to the normally developed vein system. We thought a diligent fluoroscopic observation may visualize the connections, and to elucidate further radiological details about the flow and connections.

To date, several underlying genetic factors^8^ and triggering or promoting molecular ^9,10^ or rheological processes in some types of vascular malformations or varicose veins (e) have been reported. In the development of venous malformation to form the typical shape of multiple balloons, some rheological condition may be required but not much has been discussed.

Direct injection cisternography is a commonly used radiological method for diagnosing venous malformation, and also for evaluation of cisterns and communicating vessels before sclerotherapy.^11–13^ We conducted a single arm prospective cohort study to re-evaluate the fluoroscopic findings by means of cine mode cisternography with continuous injection until optimal visualization of communicating vessels was achieved. We also inspected the existence of nearby or surrounding muscles that might act as a rheological stressor, and relationship of the radiographic findings and the treatment outcome.

## Materials and methods

We studied 30 patients who were suspected of venous malformations in the shoulder, head and neck, and scheduled to undergo direct injection cisternography prior to sclerotherapy, between 2000 and 2010. They consisted of 14 men, 16 women; age range, 5–86 years; median age, 33 years and had total, 49 lesions. All were in perfect health status except for lesions of venous malformation and no underlying abnormality was found in routine haematological studies prior to the cisternography. The definitions of venous malformations were according to the 2003 ISSVA (International Society for the Study of Vascular Anomalies) classification.^14,15^

All lesions were previously examined with ultrasonography and/or computed tomography (CT) and/or magnetic resonance imaging (MRI) studies to confirm the presence of features considered typical of venous malformation: clearly margined hypoechoic area on brightness mode (B-mode) ultrasonography and no distinguishable high-flow signal from the background on power Doppler mode, soft tissue attenuation and occasional calcification on CT, low-to-moderate signal intensity on T1-weighted images (WI) and various signal intensities on T2WI^16,17^ on MRI.

Informed consent was obtained from all patients prior to cisternography and sclerotherapy. Biopsy was not performed because of the risk of haemorrhage.

The procedures followed were performed at our institution as routine medical practice, and were in accordance with the ethical standards on human experimentation (institutional and national) and with the principles of the Helsinki Declaration. The study was approved by our institutional Ethics Committee.

### Direct puncture and cisternography

After local anaesthesia by subcutaneous injection of 1% lidocaine hydrochloride, the lesion was directly punctured with a 24- or 26-gauge plastic cannula needle (set of inner needle and plastic outer cannula). Leakage of blood from the cannula indicated successful puncture. After evacuation of the lesion by manual compression and drainage through the cannula, contrast medium (Iopamidol 300:300 mg iodine/mL) was injected under subtraction fluoroscopic observation. Injection was stopped if the patient complained of pain.

To enable clear visualization of the communicating channels, injection speed was controlled manually to maintain appropriate opacification of the lesion. Injection was continued until optimal visualization was achieved. Images were captured at two or three frames per second. The best images were expected during the first injection of contrast material because retained contrast material had a masking effect. Filling of the multilocular cavity to reveal a ‘bunch of grapes’ appearance^18^ indicated a radiological diagnosis of venous malformation.

### Classification of communication pattern

We classified communication patterns between cisterns and systemic veins according to the system of Dubois *et al.*^19^: Type 1, no visible drainage; Type 2, draining into a normal venous system; and Type 3, with abnormally ectatic draining veins.

### Identification and role of neighbouring muscle

We inspected that whether muscles are existing around the close vicinity of, or surrounding the lesion using CT, MRI and ultrasonography. Additional physical tests were conducted to examine whether lesion size was affected by gravity or by intentional contraction of nearby or surrounding muscles such as masseter.

### Sclerotherapy

Ethanol sclerotherapy to venous malformation was indicated for management of pain, bleeding and disfigurement by the lesion. While there is still remaining contrast medium in the cavities, an absolute ethanol was carefully injected through the plastic cannula into the lumen under fluoroscopy. Gradual filling with the ethanol was monitored. In our institution, the dose of ethanol was tentatively limited to less than 1 mL per 1 cm^2^-surface area of the lesion to avoid excessive injection. The injection was ended when the ethanol spread over the target lesion, or when the patient complained of pain. No further ethanol injection was added once it was ended. When the ethanol dose was thought to be insufficient a further injection was considered after four weeks later. After the ethanol injection, 300–500 mg acetaminophen tablet was given on pain.

### Evaluating therapeutic effect

Therapeutic effects were evaluated four weeks later, and which was classified into three categories: excellent, disappearance of complete (100%) or over 90% area of the malformation; partial, shrinkage of 50% or more; and weak: less than 50% or not precisely evaluable.

## Results

### Information about history and symptoms

All patients had a history of slow-growing mass that had developed over a period of years. The symptoms were apparent mass in all 30 patients, occasional pain in 12, bulging on muscular exercise including mastication in six, posture-dependent bulging in three and haemorrhage in two. Two masses developed during pregnancy.

On physical examination, all masses were soft, non-palpitating, easily collapsible by manual compression and spontaneously refilled when relaxed. Three appeared as bulging masses on the cheek during mastication. There were 21 lesions in the oral area, 18 facial lesions, two loci and three fused multiple lesions in the neck and five lesions on the shoulder/hand. Excluding the three fused multiple lesions, the diameter of the 46 lesions was 21.7 ± 10.5 mm (mean ± standard deviation), ranging from 6.0 to 48.0 mm. No flow signals were detected in the mass by power Doppler ultrasonography.

### Findings in continuous direct injection cisternography

**Figure 1:**
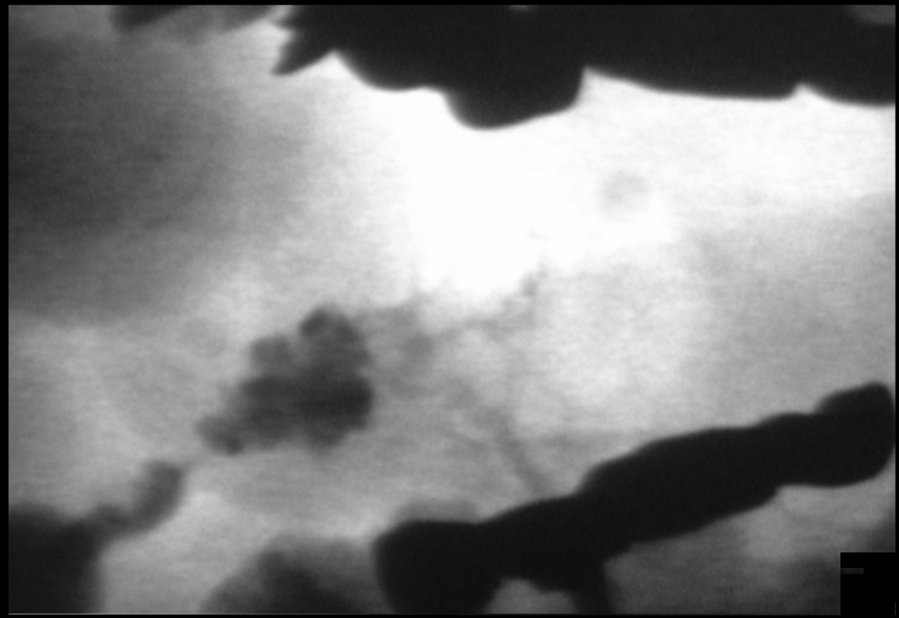
Continuous direct injection cisternography in a 65-year-old man with a lingual venous malformation located on the superior longitudinal lingual muscle. The ‘bunch of grapes’ appearance is apparent

**Figure 2:**
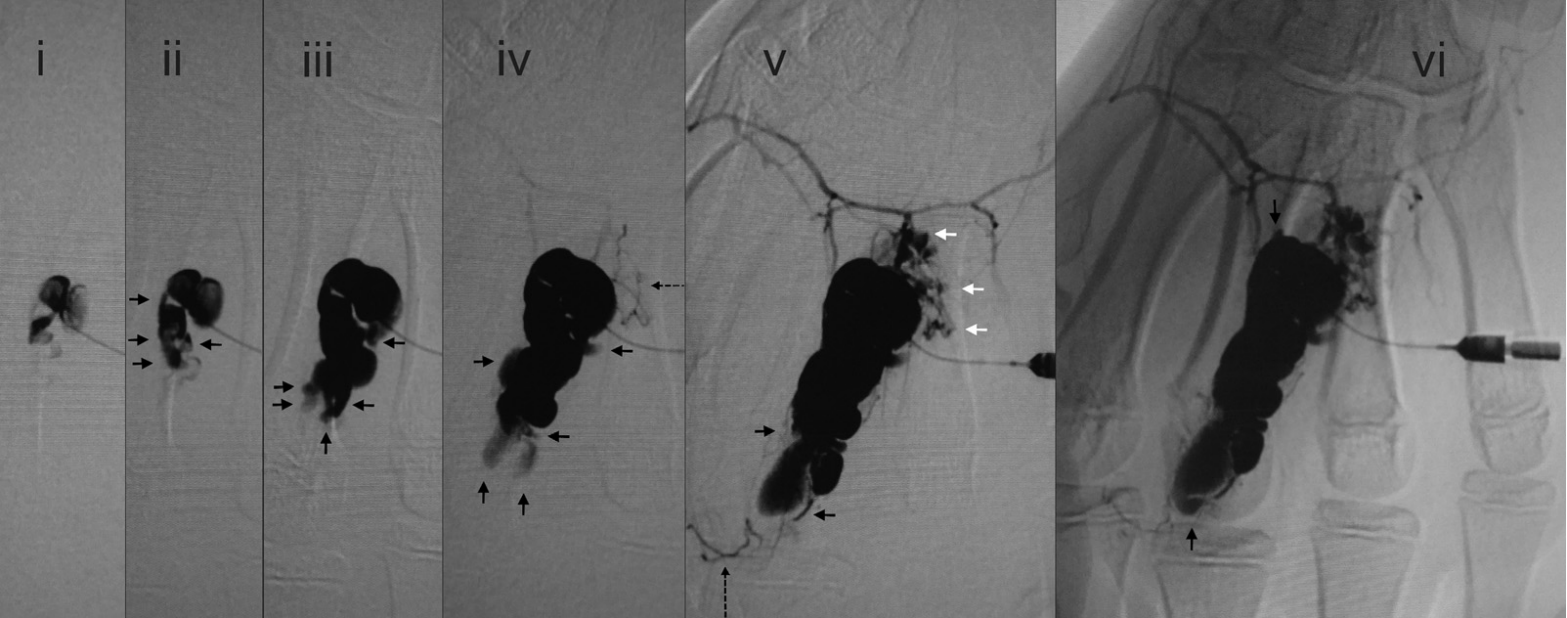
Sequential cisternal filling. Serial direct injection cisternograms (a–f) of a 13-year-old boy who complained of hand pain on exercise show the progressive appearance of cisterns and draining veins after the filling of several cisterns including satellite lesions. The initial injection filled several cisterns, with further serially filling of additional cisterns. Symbols: black arrows, newly emerged cisterns; dotted long arrows, draining veins; and white arrows, newly emerged satellite cisterns

**Figure 3:**
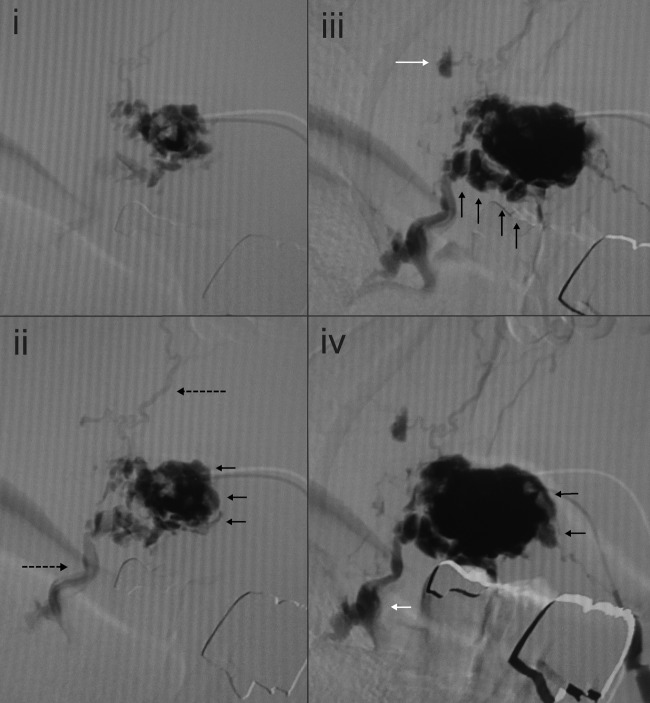
Serial direct injection cisternograms (i–v) of a soft and bulky purple mass on the inside cheek of a 67-year-old man. Two adjacent lesions were observed (a, b). In (a), an ectatic tortuous and partially bulging draining vein (Type 3) [dotted arrow in (ii)], and satellite cistern [white arrow in (iii)] appear after initial filling of the punctured cistern. In (b), a draining vein [dotted arrow in (v)] appears after filling of the cistern (Type 2B). Symbols: black arrow, newly emerged cistern; dotted arrow, draining vein; white arrows in set a, satellite cisterns

**Figure 4:**
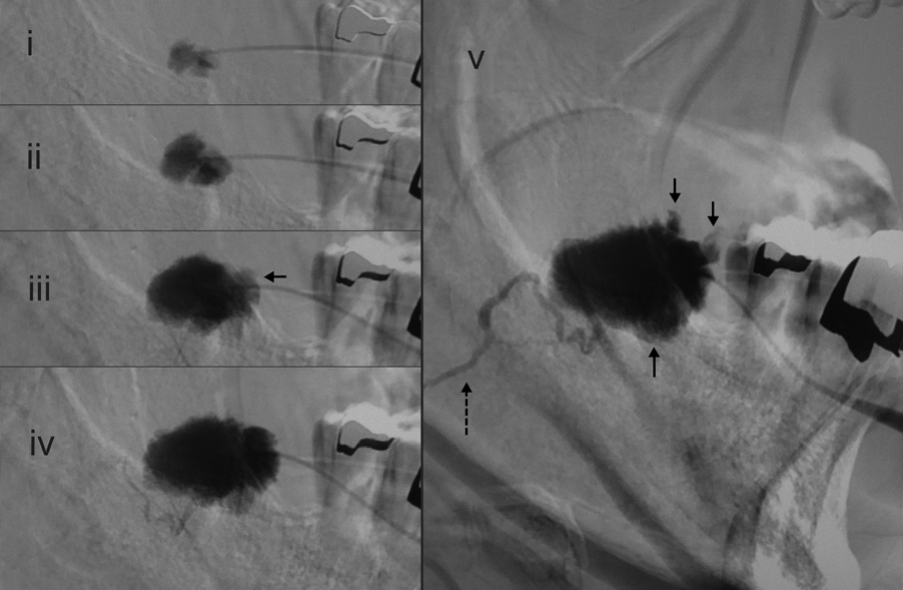
Serial digital subtraction images during continuous injection of a cheek mass that swelled during mastication, in a middle-aged woman. Serial filling of the cisterns is observed (a–c). Draining veins appeared after filling of the cisterns (Type 2B). Unsubtracted images (d–f) obtained during pauses in injection show that the draining veins disappeared within seconds but the cisterns remained filled, showing Type 1 appearance. Plain CT images show calcification in the lesions in the left masseter muscle (g) and sustained pooling inside the fascia of the masseter muscle 4 hours after injection (h). Symbols: black arrow, newly emerged cistern; dotted arrow, draining vein

**Figure 5:**
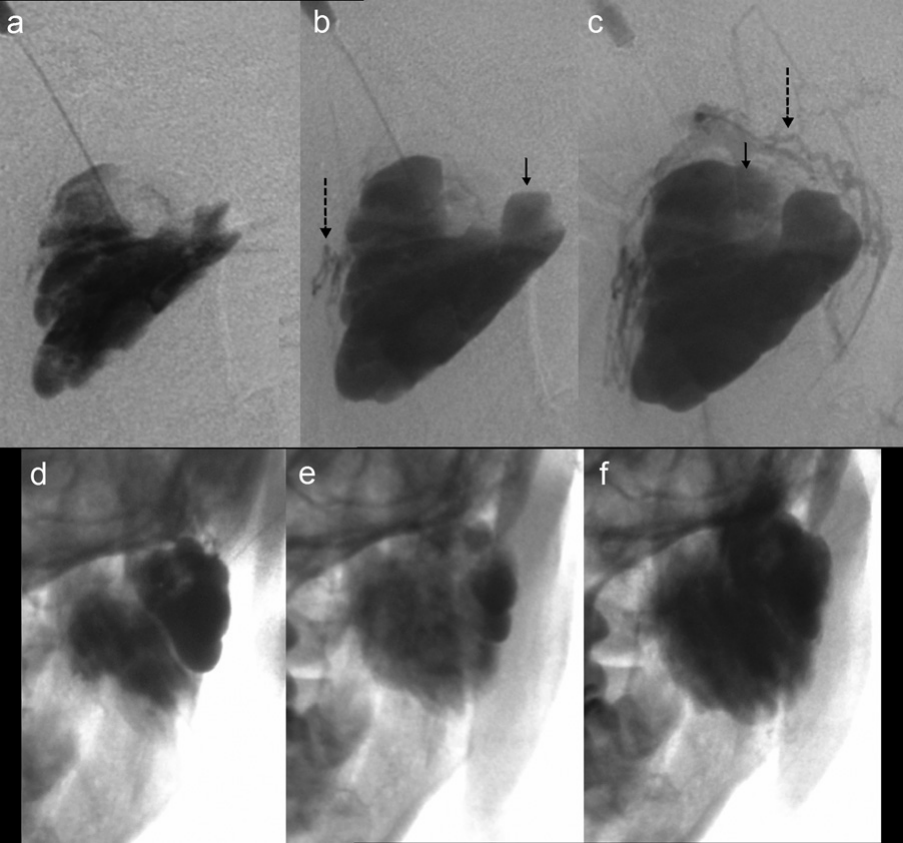
Intramuscular and extramuscular cisterns. Cisternography in a young woman who began experiencing pain in the upper extremity at the end of the first trimester. The ‘bunch of grapes’ pattern is seen around the clavicle (a), and an elongated cystic pattern is visible in and along the fascicles in the pectoralis major muscle (PM) (b). Appearance of the drainage vessel was faster at the PM aspect (not shown: continuous injection was terminated because of pain). MRI images (c–e) show continuity of the cisterns, with subclavicular cisterns showing continuity with those on the trapezius muscle and the PM. Symbols: white arrow, cisterns in the PM; dotted white arrow, cisterns in the trapezius muscle

Each direct puncture cisternography procedure was successfully accomplished within 30 minutes. Injection rate ranged from 0.5 to 1.0 cm^3^ per second, and filling was completed within 5–20 seconds depending on the size of the lesion and the injection rate. There were no examination-related adverse events. The hallmark ‘bunch of grapes’ appearance was confirmed in all lesions (Fig. 1, also Figs 2–5).

### Serial appearance in cisternography

In all lesions, the cisterns filled sequentially; i.e. from the initially injected cistern(s) to the neighbouring cistern(s) (Fig. 2). The cistern injected first was connected either to one other cistern, or more commonly to several other cisterns. In six lesions, small satellite bunches were visible leading off the draining veins after filling of the main cisterns (Figs. 2 and 3). The ‘bunch of grapes’ appearance remained as areas of sustained pooling after continuous injection was stopped (Fig. 4). It was difficult to perfectly track the all course of the appearance due to fluoroscopic masking effect by pooled contrast media.

### Appearance of draining veins

In all lesions, draining veins were clearly visualized during continuous injection (Figs. 1–5; Table 1). The timing of their appearance varied. The earliest observation was immediately after the initial filling (Fig. 3a) and the latest was after filling of several cisterns (Figs. 2, 3b and 4). In the latter, we were unable to identify direct branching of the cistern from the draining vein. Contrast medium flushed quickly from the draining veins immediately after injection was stopped, whereas cisternal pooling persisted (Fig. 4e–g). Two lesions had a rather ectatic draining vein that emerged early (Fig. 3a) and was classified as Type 3. There were no large ectatic draining veins (varicose veins) in the present subjects.

**Table 1:** Symptoms

Symptoms	Count
Apparent mass	30
Occasional pain	12
Bulging on muscular exercise	6
Posture-dependent bulging	3
Haemorrhage	2
Total (overlapped)	53

### Relationship to the muscles

Of 49 lesions, 48 were located in and/or on muscle tissue (Figs. 4b and 5; Table 2). One lesion at appeared extraction space of upper molar tooth had no muscles in the vicinity. Nineteen predominant lesions were suspected to be intramuscular. In one lesion located in the pectoralis major muscle, extension of cisterns was observed in an interfascicular pattern, suggesting their location in the perimysium, the interfascicular loose connective tissue space that contains blood vessels and nerves (Fig. 5). All lingual lesions had an intrafascial component beneath the aponeurosis linguae fascia that covers the lingual surface. Protrusion-during-massetication-type venous malformations occurred in/on the masseter and orbicularis oris muscles in juvenile patients (range of age, 12–15 years) (Table 3).

**Table 2:** Incidence of draining patterns

Type		Subtype	Count	
I	No visible drainage		0	
II	Draining into a normal venous system	Early appearing	20	47
		Late appearing	17	
III	Abnormally ectatic draining veins	Small	2	
		Large (varicose)	0	
Total			49

**Table 3:** Muscles associated with lesions evaluated in the present study

Region		Site of lesions	Muscle name	Predominant order of muscle locative relation			Muscle unidentifiable
				First	Second		
Head and neck							
	Oral cavity						
		Tongue	Superior longitudinal	11 (11)			
		Oral floor	Inferior lingual	3			
		Palate	Palatepharyngeal	3			
			Masseter	3 (3)	2 (2)		
		Tooth extraction space					
			(Unidentifiable)				1
	Face						
		Lip/Perioral	Perioral	13			
			Depressor labi	2	2		
		Bucchal	Bucchinator	3	3		
	Neck						
		Lateral	Sternocleidomestoideus	2			
		Huge lesion	Fused multiple in one site	3			
Shoulder/Trunk			Pectoral major	1 (1)			
			Trapezius	2 (2)			
Hand			Flexor policis and adductor policis	1 (1)			
			Interossei palmaris and dorsalis	1 (1)		total	
			Total	48 (19)	7 (2)	55 (21)	1

The number of intramuscular lesions is given in parentheses.

### Results of sclerotherapy

Sclerotherapy was completed in all 46 Type 2 lesions, but for the Type 3 lesions the treatment was limited to partial. Mass reduction effect was excellent for all Type 2 lesions, and partial in all Type 3. All patient obtained symptomatic improvements: there was no occasional pain due to the venous malformation, no bulging on muscular exercise including mastication, no haemorrhage after the treatment. Posture-dependent bulging was also disappeared at the treated site and weakly effective even at the vicinity untreated lesions in the three huge lesions.

During the follow-up period over of 6.5 years (range, 2–9 years), there has been no further episode of pain or haemorrhage after the treatment in all patients, no bulging on muscular exercise or posture-dependent bulging except the huge case. One lesion relapsed was located at tooth extraction space which had no vicinity muscles.

## Discussion

The aetiology of venous malformations remains still mysterious. To date, the venous malformation is thought as an embryonic vascular structure remnant along the venous system development^1–5^ so that its outflow/drainage should be all connected to the normally developed vein system, and their growth might be stimulated or promoted afterward.

### Visualization of communication channels

Continuous injection revealed communication channels with systemic veins in all lesions; after injection was stopped, the contrast material flushed out and most of the draining veins became invisible, leaving only residual contrast in the cisterns as typically shown in Fig. 4 and in other reports.^13^ This suggest some lesions conventionally classified as Type I^19,20^ with radiographically solitary cisterns might have shown fine or moderate communication channels.

### Small satellite lesions

We found several small satellite lesions visualized via connected veins in several of present cases, where some were close to the main bunch and others apart (Figs. 2 and 3), as parts of a cluster. The existence and distribution might be interpreted as reflecting that of the remnants along the venous system. There might be other satellite lesions not visualized.

### Serial appearance of cisterns

**Figure 6:**
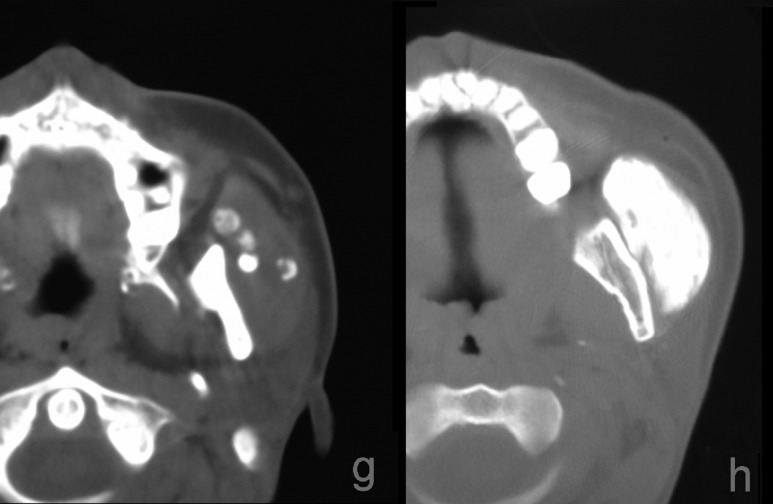
Postulated serial filling patterns. When a dilated cistern intercepts the inflow, a stepwise filling of one to the next occurs (a). When dilated cisterns are located on the side of the vessel wall, filling occurs almost at once if the distance (d) is short (b). Intermediate, extreme and mixture patterns can be variously postulated

The cisterns appeared serially one to another or, to several others. It was difficult to perfectly track the fluoroscopic appearance; instead, we postulated possible patterns of cistern filling to explain viciousness of the seriality (Fig. 6).

### Imaginary consideration on rationale of muscles

The development of ballooning disfigurement of cisterns may be facilitated by many factors. Muscles are well known to function as a pump: lesions in the masseter muscle inflated during eating of the juvenile. Frequent incidence of intramuscular venous malformations has been reported previously.^21^ All lesions we observed were located in or on the muscles. Though it is still imaginary, but we may speculate that rheological stress originating from the muscle functioning as a pump may be one of the important facilitating factors and which wanes with weakening of muscles.

## Conclusion

The present study suggested that communications from venous malformation to the systemic vein are fluoroscopically confirmable. These radiographic findings were thought explainable in relation to developmental nature or facilitating process of venous malformation.
